# Sacral neuromodulation for refractory ulcerative colitis: safety and efficacy in a prospective observational series of eight patients

**DOI:** 10.1007/s10151-023-02793-3

**Published:** 2023-04-12

**Authors:** Farouk Drissi, Arnaud Bourreille, Michel Neunlist, Guillaume Meurette

**Affiliations:** 1 Chirurgie Cancérologique Digestive et Endocrinienne, Institut des Maladies de l’Appareil Digestif, University Hospital of Nantes, Hôtel Dieu, 1 Place Alexis Ricordeau, 44093 Nantes, Cedex 01, France; 2 The Enteric Nervous System in Gut and Brain Disorders, Institut des Maladies de l’Appareil Digestif, University of Nantes, Inserm, TENS, Nantes, France; 3 University Hospital of Nantes, Hôtel Dieu, Nantes, France; 4 Division of Digestive Surgery, University Hospital of Geneva, Geneva, Switzerland

**Keywords:** Sacral neuromodulation, Ulcerative colitis, Intestinal epithelial barrier permeability, Inflammation

## Abstract

**Purpose:**

Ulcerative colitis (UC) treatment is mainly based on immunosuppressive therapy. As anti-inflammatory effects of sacral neuromodulation (SNM) have been previously reported in animal models, we conducted a pilot study aimed at assessing clinical, biological, and endoscopic response but also safety of SNM use in UC refractory to medical therapy.

**Methods:**

Adult patients with histologically proven UC resistant to immunosuppressive therapy were invited to enroll in the study. Primary outcome was the rate of UC remission (UCDAI score ≤ 2, without any criteria > 1) at 8 weeks (W8). Secondary outcomes were biological and endoscopic response also evaluated at W8 and W16. Subsequently, every patient was followed every 6 months. Adverse events were prospectively collected for safety assessment during the follow-up.

**Results:**

Eight patients, with mean age 47 years old, suffering from UC for 2–13 years were included. There were no complications in relation to SNM procedure. The acceptance of the device was excellent in all patients. Clinical and endoscopic remission was obtained at W8 in one patient (12.5%) and three other patients (37.5%) were responders at W16. At review (mean follow-up of 4 years), two patients (25%) were in remission and two (25%) were responders.

**Conclusion:**

SNM application is safe in patients suffering from refractory UC. Effects on disease activity were mainly observed after 16 weeks. Larger prospective studies are mandatory, but SNM could be a way to reinforce medical therapy and reduce the use of immunosuppressive drugs.

## Introduction

Ulcerative colitis (UC) is an inflammatory bowel disease that along with Crohn’s disease affects rectal and colonic mucosa. The precise inflammation trigger remains unknown but it is highly suspected that an inappropriate immune response to the microbiota is involved, in addition to other environmental factors in genetically susceptible subjects [1]. Local intestinal inflammation is sustained by an increase of proinflammatory cytokines such as interleukin (IL)-1β, IL-6, and tumor necrosis factor (TNFα). UC treatment is mainly based on immunosuppressive drugs some of which are targeted against these inflammatory proteins. Surgery is considered in case of medical treatment failure and/or acute severe colitis, e.g., for approximately 10% of the patients 10 years after diagnosis [2].

Sacral neuromodulation (SNM) is a validated technique in the treatment of fecal incontinence through stimulation of S2 and/or S3 nerve root by an electrode implanted nearby [3]. A permanent low-amplitude stimulation is then delivered by a pulse generator. Among the different mechanisms of action of SNM, the parasympathetic pathway is among the demonstrated effects through the vagus nerve, exerting anti-inflammatory effects [4]. In fact, previous experimental models of SNM in colonic inflammation showed an improvement of mucosal healing in stimulated animals [5]. Moreover, in a single-patient experience, Brégeon et al. reported an improvement of fecal incontinence and disease activity scores in a woman with persisting disabling symptoms of UC despite full medical treatment [6].

In this study, we aimed to prospectively assess the safety and clinical, biological, and endoscopic response to SNM in a cohort of patients with UC refractory to medical therapy.

## Methods

We conducted a monocentric pilot study, including adult patients with histologically proven UC from at least 1 year. Patients were eligible if they were diagnosed with (i) UC at most extending to the splenic flexure (E1 or E2 of the Montreal classification), (ii) resistant to immunosuppressive therapy (including azathioprine, 6-mercaptopurine, methothrexate, or anti-TNFα) for at least 8 weeks without recent modification of the treatment; with Ulcerative Colitis Disease Activity Index (UCDAI) > 5 (endoscopic subscore ≥ 2). Exclusion criteria were (1) patients aged under 18 years, (2) severe complications requiring inhospital intravenous therapy or immediate surgical treatment, (3) infectious proctitis or colitis, (4) intravenous steroid therapy, (5) prednisone > 20 mg/day, (6) pregnancy.

Patients underwent a 3-week test period after tined lead positioning as routinely performed for incontinent patients. If no adverse event occurred, a permanent InterStim™ neurostimulator system was implanted. Settings used in patients with fecal incontinence were applied.

Outcomes were measured at baseline (W0), week 8 (W8), and week 16 (W16). Patients were then regularly followed with clinical assessment every 6 months. The primary outcome measure was the rate of UC remission (UCDAI score ≤ 2, without any criteria > 1) at W8. Secondary outcomes measures were (a) response (decrease ≥ 1 point of the physician rating of disease activity and ≥ 1 point of the three other items of the UCDAI without increase in the other dimensions of the score), (b) clinical response (decrease ≥ 3 points of the UCDAI clinical items), (c) endoscopic response (decrease ≥ 1 point of the UCDAI endoscopic item), (d) biological response (decrease of the C-reactive protein and/or fecal calprotectin levels), (e) adverse events.

Intestinal epithelial barrier permeability was assessed from rectal biopsies collected at W0, W8, and W16 during endoscopic evaluation. Rectal mucosa was dissected and mounted in dedicated Ussing chambers. The gradient of change in fluorescence intensity at the basolateral side allowed one to determine the paracellular permeability.

Patients provided informed consent. The study was approved by the ethics committee (No. 16/08–1006).

Hypothesis and sample size: To show modifications of UCDAI after 16 weeks with remission induction by 50%, and assuming a dropout rate by 20% due to infection or adverse effects during the test phase, we calculated that 10 patients were to be included in the study; after inclusion of eight patients without dropout, the study recruitment was stopped.

## Results

Eight patients, with mean age 47 ± 16 (22–71) years old, suffering from UC lasting for 2–13 years were included (Table 1). The test phase of SNM was uneventful for every patient and definitive implantation was achieved 3 weeks later in all cases. Median final programming settings were amplitude 1.09 V, frequency 14 Hz, and pulse width 210 µs.

**Table 1:** Patient characteristics and outcomes

Patient	Age	Gender	Treatment at inclusion	Treatment modification	Status at W16	Adverse events	FU duration	Status at last FU	Stimulation
1	27	F	Azathioprine	+	Non-responder	Shoulder pain, rash, UC flare	6	Remission	On
2	50	F	5-ASA	−	Remission	–	6	Remission	On
3	62	M	Cortancyl	−	Responder	–	5	Death	Off
4	36	M	Azathioprine	−	Non-responder	–	4	Non-responder	On
5	61	M	5-ASA	−	Responder	Back pain	3	Responder	On
6	38	F	5-ASA	−	Non-responder	Lead disconnection, gastroenteritis Intrauterine fetal death	3	Non-responder	On
7	56	M	5-ASA	+	Responder	Small bowel diverticulitis	3	Non-responder	Explanted
8	73	M	5-ASA	+	Non-responder	–	3	Responder	On

Age and follow-up duration are expressed in years. Adverse events, status, and stimulation at last follow-up are detailed for each patient. The need for treatment modification before W16 is specified as + (yes) or − (no)

*FU* follow-up,* 5-ASA* 5-aminosalicylic acid,* UC* ulcerative colitis

### Safety assessment

Accidental disconnection of the electrode occurred during the test phase in one patient but no other major complication related to SNM was reported. Device acceptance was excellent in all patients. Three serious adverse events were subsequently reported: ulcerative colitis flare (one patient), diverticular small bowel perforation (one patient), and one intrauterine fetal death (unknown pregnancy at the time of the procedure). Minor adverse events occurred in four patients: one shoulder pain, one cutaneous rash, one back pain, and one gastroenteritis. No relation was established between SNM procedure and the occurrence of adverse events. The outcome was favorable in every patient.

### Efficacy on UC

**Fig. 1:**
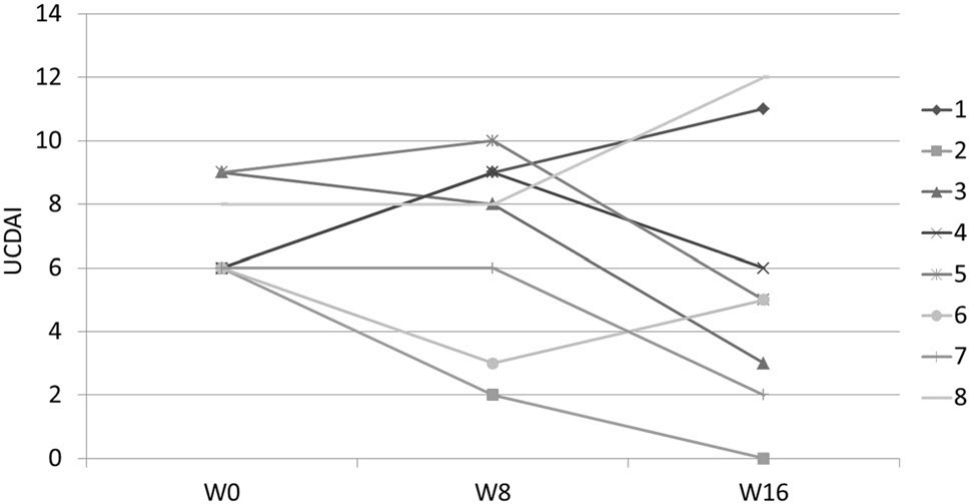
Effects of sacral neuromodulation on Ulcerative Colitis Disease Activity Index (UCDAI) at inclusion (W0), 8 weeks post-implantation (W8), and 16 weeks post-implantation (W16)

**Fig. 2:**
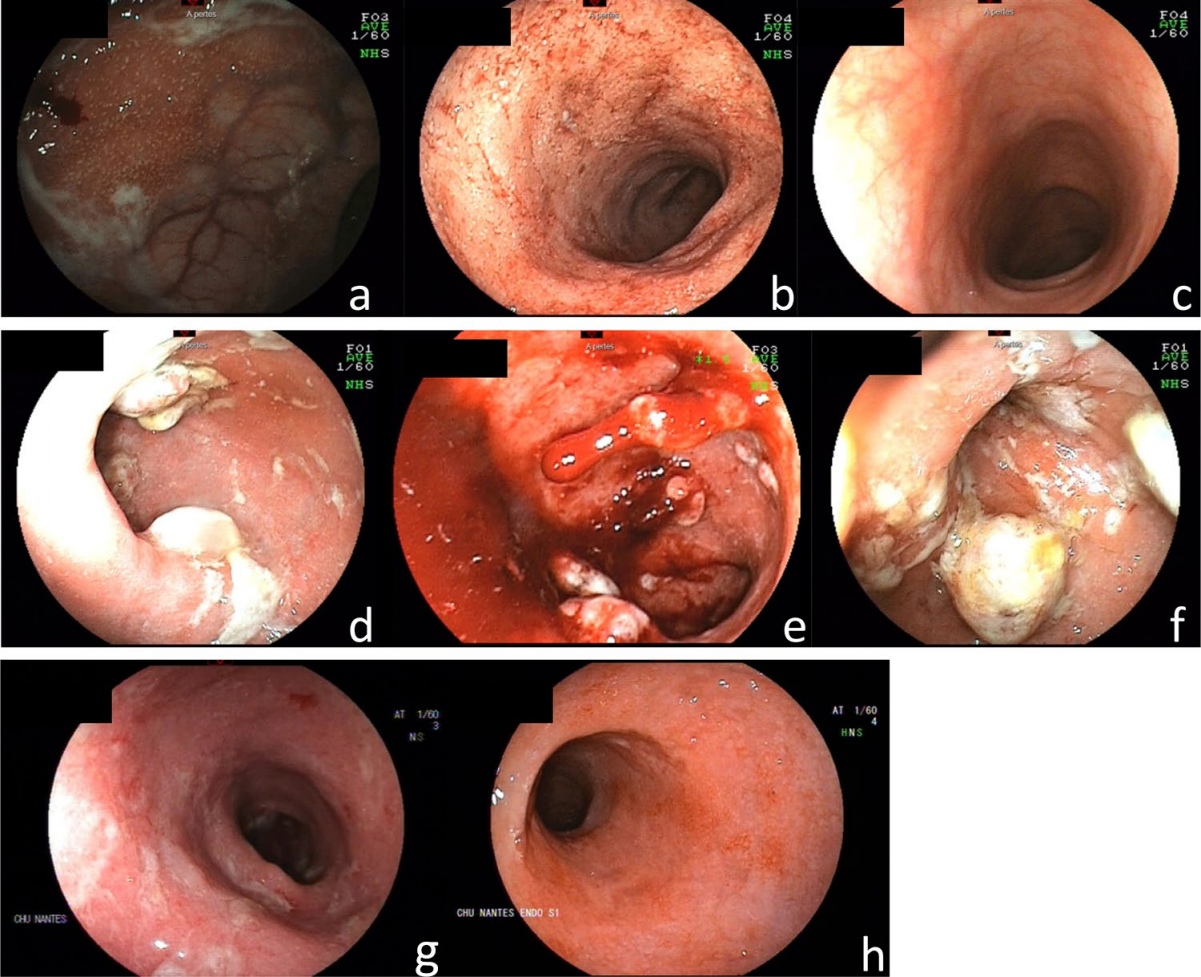
Endoscopic evaluation of ulcerative colitis. Patient 1 at baseline (**a**), W16 (**b**), and 3 years (**c**). Patient 4 at baseline (**d**), W8 (**e**), and W16 (**f**). Patient 7 at baseline (**g**) and W16 (**h**)

**Fig. 3:**
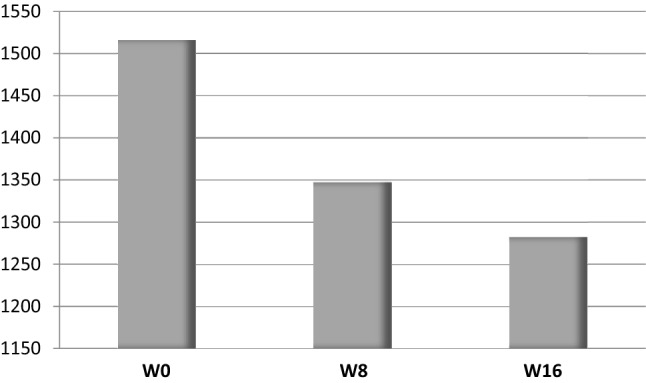
Effects of sacral nerve stimulation on fecal calprotectin levels (µg/g) at inclusion (W0), 8 weeks post-implantation (W8) and 16 weeks post-implantation (W16)

**Fig. 4:**
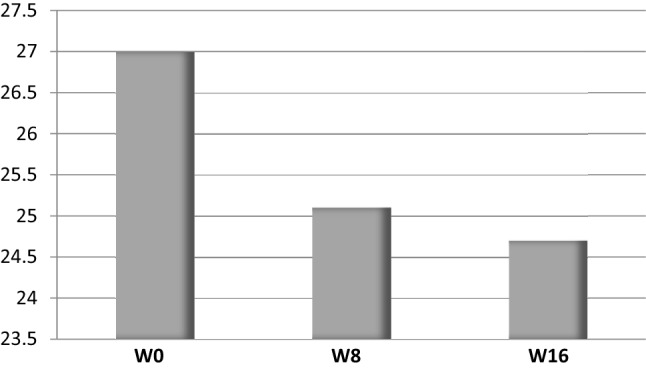
Effects of sacral neuromodulation on paracellular permeability (a.u.) at inclusion (W0), 8 weeks post-implantation (W8), and 16 weeks post-implantation (W16). Paracellular permeability is quantified as the area under curve of sulfonic acid flux in the paracellular space of intestinal epithelial barrier

Clinical and endoscopic remission at W8 was obtained in one patient (12.5%) and maintained at W16 (Fig. 1). Three (37.5%) other patients were responders at W16. The rate of clinical response at W8 and W16 was respectively 25% and 37.5%, whereas endoscopic response was observed in 50% of the patients at W16 (Fig. 2). Bleeding completely disappeared in 3 (37.5%) and 5 (62.5%) patients at W8 and W16, respectively. Fecal calprotectin levels decreased from 1516 (227–2000) to 1282 (40–2207) µg/g (Fig. 3). Quality of life, stool frequency, stool consistency, and Wexner score remained unchanged in the whole cohort despite heterogeneity between the patients. After analysis of biopsies, we found a decrease of the paracellular permeability at W8 and W16 (Fig. 4). Histological response was achieved in only one (12.5%) patient at W16.

After long-term follow-up, on average 4 years post-implantation, 6/8 (75%) patients were still treated with SNM. Among them, two patients were in remission (without need for additional treatment), two were responders (further introduction of adalimumab in one patient), and two were non-responders with stable disease activity. The two remaining patients definitely failed SNM in the long-term and thus the stimulator was explanted or turned off and biologics were then started. Explantation was uneventful with complete removal of the lead. One of these patients required complementary coloproctectomy.

## Discussion

This is the first study aiming to assess the therapeutic effect of SNM in a cohort of patients suffering from UC. We first showed that SNM use in patients with UC was safe and feasible as we did not have any device- or procedure-related adverse event following the SNM implantation. Despite that, we could not demonstrate that SNM was an effective treatment for inducing remission after 8 weeks. However, after 16 weeks of SNM, a response (3/8) or remission (1/8) could be achieved in half of the patients.

We also observed an endoscopic response in four patients at W16. In particular, two patients retrieved normal mucosa. The downstaging of mucosal lesions in half of the patients at this stage is encouraging as endoscopic response predicts long-term remission [7].

No clear benefit in terms of stool frequency, stool consistency, abdominal comfort, and quality of life could be demonstrated. An explanation could be the heterogeneity of the cohort regarding stool frequency and abdominal discomfort. The impact of UC on abdominal comfort and quality of life at baseline was mild, as stool frequency ranged between 1 and 5 per day.

In a previous case report, we performed SNM in a patient with refractory proctitis and UC [6]; fecal continence and disease activity index were significantly improved with results sustained over time. Moreover, rectal barrier permeability was decreased and expression of junctional proteins was increased. In a rodent model, Tu et al. demonstrated that SNM exerted anti-inflammatory effects via an afferent spinal–efferent vagal pathway [4]. The expression of pro-inflammatory cytokines was decreased whereas secretion of anti-inflammatory proteins was increased.

The long-term continuation of SNM in 6/8 patients, without adverse effect, is also encouraging. One of these patients required the introduction of adalimumab during follow-up to maintain the response. In the need for treatment escalation, the impact of SNM parameter variation has to be evaluated since it has been shown that the modification of pulse width and frequency also influences the efficacy of SNM on colonic inflammation [8]. The exact position of SNM in refractory UC needs to be clarified but it could be proposed in the case of immunosuppressive drugs failure, in order to potentially reinforce their efficacy, particularly before indicating radical surgery.

Even if it is a small series, without control group, this is the first one to assess the therapeutic effects of SNM in patients with UC refractory to immunosuppressive therapy. These results should be confirmed in a larger randomized controlled trial.

## Conclusion

This work demonstrates that SNM can be safely used in patients with UC. SNM mainly exerted late effects on the response after 16 weeks. Hereby we provide encouraging data for further exploring SNM use in UC, in combination with immunosuppressive therapy, in order to reinforce the benefit of medical therapies or reduce their use.

## Data Availability

All data supporting the findings of this study are available within the paper. Additional informations are available from the corresponding author upon reasonable request. Data are located in controlled access data storage in our dedicated research unit at the University Hospital of Nantes.
